# A Real-Time Electrical Load Forecasting in Jordan Using an Enhanced Evolutionary Feedforward Neural Network

**DOI:** 10.3390/s21186240

**Published:** 2021-09-17

**Authors:** Lina Alhmoud, Ruba Abu Khurma, Ala’ M. Al-Zoubi, Ibrahim Aljarah

**Affiliations:** 1 Department of Electrical Power Engineering, Faculty of Engineering Technology, Yarmouk University, Irbid 21163, Jordan; lina.hmoud@yu.edu.jo; 2 King Abdullah II School for Information Technology, The University of Jordan, Amman 11942, Jordan; rubaabukhurma82@gmail.com (R.A.K.); or alzoubi@correo.ugr.es (A.M.A.-Z.); 3 School of Science, Technology and Engineering, University of Granada, 18011 Granada, Spain

**Keywords:** artificial neural network, hourly demand, load forecasting, maximum demand, total demand

## Abstract

Power system planning and expansion start with forecasting the anticipated future load requirement. Load forecasting is essential for the engineering perspective and a financial perspective. It effectively plays a vital role in the conventional monopolistic operation and electrical utility planning to enhance power system operation, security, stability, minimization of operation cost, and zero emissions. Two Well-developed cases are discussed here to quantify the benefits of additional models, observation, resolution, data type, and how data are necessary for the perception and evolution of the electrical load forecasting in Jordan. Actual load data for more than a year is obtained from the leading electricity company in Jordan. These cases are based on total daily demand and hourly daily demand. This work’s main aim is for easy and accurate computation of week ahead electrical system load forecasting based on Jordan’s current load measurements. The uncertainties in forecasting have the potential to waste money and resources. This research proposes an optimized multi-layered feed-forward neural network using the recent Grey Wolf Optimizer (GWO). The problem of power forecasting is formulated as a minimization problem. The experimental results are compared with popular optimization methods and show that the proposed method provides very competitive forecasting results.

## 1. Introduction

Power system planning is indispensable. It starts with a forecast of expected future load requirements on both demand and energy. Demand forecasts are used to determine the capacity of generation, transmission, and distribution. Estimating both demand and energy requirements is crucial to effective system planning. A load forecast can help establish the policy for the procurement of capital equipment and fuel. Forecasting plays a significant role in planning and saving in the cost. Further energy forecasts are needed to determine the future of fuel requirements. When the fuel prices soar high, the rate relief to maintain an adequate return rate can also be predicted by load forecasting. Load forecasting supports an electric utility to make essential decisions such as purchasing and generating electrical power, load switching, and infrastructure development. Thus, this study helps to minimize the gap between demand and supply of power to avoid shortage or surplus in the power system. Forecasting is considered a risky business. It is a mechanism of extrapolating the past data into the future using a mathematical formula or gathering the expert’s trends. Load forecasting in a power system is a technique used by the utility that provides the power to predict the energy needed to meet demand and supply equilibrium. It is mainly based on area, location of load, type of load (industrial, commercial and residential) [1,2,3].

The electrical power grid challenges, such as the vulnerability assessment, is a computationally-intensive process; the measurements are noisy, the uncertainty in the operating conditions, and the grid needs to be continually updated. The energy management system manages the operating of the electrical grid within the safe limit. It is automatically adjusted to demand, identify risk, take preventive blackouts action, expedite restoration and buy energy from the neighboring grid or not during the day. However, there is no right way of designing features. The distribution companies face discrepancies between the expected and actual energy production, leading to unbalanced grid energy shortages and surpluses. The lack and excess are costly for utilities as they may cause making energy. Therefore, the utility company must make an informed a balancing decision to minimize costs and operational complications. The more accurately the energy produced from can be predicted. Energy generation must be accurately forecasted to prevent energy shortages and surpluses. Utility companies must be able to make informed grid balancing decisions to minimize cost and operational complications. One reason for these companies’ low revenue is the lack of accurate demand forecast and demand planning.

Load forecasting is considered a hot topic across energy companies. Thus it can be defined as the estimation for future load by an industry or utility company. This estimation of future electricity demand both affects the strategic and operational level of decisions in the industry. Precise load forecasting helps the electric utility make commitment decisions, reduce spinning reserve capacity, and schedule the maintenance plan properly. Load forecasting can be used for forecasting the operational assessment to minimize cost and operational effects while maximizing revenues. The main steps to develop a forecast can be summarized as follows: (i) determining the primary use of the forecast; (ii) identifying items; (iii) determining the time horizon; (iv) selecting the forecasting model and validating this model.

Forecast accuracy is not an end itself; it improves performance and achieves cost-saving and good planning. It is useful in load forecasting to consider the adaption period by adjusting the forecasting process to the right level with the most recent data. It is based on the comparison of the last made forecast and the actual belonging outcome. Accurate forecasting of demand helps in cost minimization.

Optimization [4,5] is finding the optimal points (maxima and minima) in a particular system. It involves testing out many different combinations of inputs to determine the resulting outputs. Optimization algorithms are much more sophisticated than enumeration. Optimization theory has custom-made algorithms to find the optimal solution with minimal computation by exploiting knowledge of the model. Optimization techniques are used to find the better and faster optimal design—besides the optimal decision evaluations, proper trade-offs, and non-intuitive analysis. This study sake to develop an automated technique for short-term load forecasting (STLF) using multi-layer perceptron neural network (MLP) [6]. It provides a quick and accurate prediction of different cases of hourly load for the next seven days at the level of accuracy required by today’s complex and competitive power market. A gray wolf optimizer (GWO) is chosen as one of the algorithms applied to Jordan’s electrical load forecasting system to find the best design, the lowest cost, and the highest efficiency. GWO has proven its impressive performance in a wide range of applications, including engineering [7], medical [4] and different machine learning applications [8]. The primary issue for the widespread usage of GWO in applications is that it has a unique hierarchical population structure. This helped GWO to save the best solutions throughout iterations. Furthermore, GWO has adaptive parameters that make a smooth transition between the exploration and exploitation phases of the optimization process. This indicates the capability of the optimizer to alleviate the entrapment in local minima. To the best of our knowledge, this is the first work on optimized load forecasting using GWO. Innovative system performance, increased system resilience and reliability, cost reduction and environmental productivity are the main criteria for measuring system performance. The requirement is valid and clean data from multiple resources to build a prediction model.

The rest of this paper is arranged as follows. Section 2 summarizes the related work. Section 3 introduces the preliminaries that are used in this research. Section 4 introduces the data collection process. The proposed forecasting method is addressed in Section 5. Experiments and results are demonstrated in Section 6. The paper finishes with the conclusion and future work in Section 7.

## 2. Related Work

Forecasting techniques can be divided into two fundamental techniques: quantitative and qualitative [9,10]. Time series [11], regression methods [12], moving average [13], exponential smoothing [14] and trend projection [15] are examples of the quantitative techniques. They are used when the situation is stable and historical data exists. Also, it involves mathematical techniques. Whereas the qualitative techniques [16], such as high-level experts [17] and Delphi methods [18]. They are used when the situation is vague and little data exists. They involve intuition experience and sometimes statistical models augment it.

STLF for Jordan power system grid based on optimized NN is demonstrated in many studies [19,20,21]. The authors in [19], proposed an STLF by adopting the NN in Jordan’s power system. Different optimization methods such as particle swarm optimization (PSO), genetic algorithm (GA), and elephant herding optimization (EHO) were applied to optimize the updated parameters. In this study, the authors computed the error before and after the optimization process was performed. The results proved that the optimization methods based on NN achieved competitive performance for load forecasting. The main information the authors obtained from the study is that a two-layer NN is the best for load forecasting. In [20], the authors developed a new method based on a rolling stochastic Auto-Regressive Integrated Moving Average with Exogenous (ARIMAX) to minimize the effect of the COVID-19 pandemic on the performance of the load forecasting model. The proposed model tried to enhance the forecast performance by identifying the non-smooth demand nature by suggesting different future demand scenarios based on a probabilistic model. The proposed model achieved better results than the ARIMAX model and Artificial Neural Network (ANN) and reduced the error of forecasting by up to 23.7%. In [21], a new method was proposed based on NN for LTLF of the Jordanian power system from 2015 to 2029. Two types of feedforward neural networks (FFNN) were adopted; the back-propagation and the radial basis function neural networks; (BPNN) and (RBFNN), respectively. The National Electric Power Company (NEPCO) dataset was used in the training and testing of the proposed NN methods. The performance accuracy of the proposed NN methods was evaluated in terms of the mean square error (MSE) and mean absolute error (MAE). The obtained results were competitive and promising. The proposed NN method can be effectively used for forecasting the annual peak loads of Jordan’s system. In [22], efficient STLF approaches in machine learning models are investigated in terms of overall execution time and scalability. A combination of three different techniques, extreme gradient boosting (XGB), light gradient boosting (LEGBM), and multilayer perception (MLP), are selected, the performance superiority is approved, including the training time [23]. A proposed model competes with the traditional ones based on modifying non-parameter kernel-density estimation to validate the prediction intervals and NSGA-II is adopted for multi-objective optimization [24]. A new STLF proposed technique based on time series and nonlinear relationship of load data hybrid with the multi-temporal spatial scale method [25]. An implementation of STLF online adaptive recurrent neural network is presented. In this work, the model is updated continuously as new data [26]. In [27], optimal electrical load forecasting is presented. A root cause of the economic reduction is discussed based on an insensitive linear-linear cost using asymmetric support vector regression of the data. A Busload forecasting is carried out directly into algorithms, thus in [28].

An enhanced cleaning data setup for quality issues, such as temporary gross errors and random noise. The accuracy is enhanced as a consequence. In [29], a logistic mixture vector autoregressive model is adopted for LF analysis. It takes into account both the pattern variation and pattern-to-pattern. The internal clustering is incorporated to enhance accuracy. STLF analysis based on the transformed data and the statistical machine learning algorithm considering the penetration of renewable energy resources is investigated in [30], a more resilient and robust grid through accurate electrical load forecasting and using the cutting edge of the machine learning techniques.

This study proposed an automated method for STLF based on NN. It provides a quick and accurate prediction of different cases of hourly load for the next seven days at the level of accuracy required by today’s complex and competitive power market. According to the No-Free-Lunch (NFL) theorem [31,32], no optimization algorithm can solve all optimization algorithms with the same level and performance. Therefore, in this study, a GWO is chosen as one of the algorithms applied to Jordan’s electrical load forecasting system to find the best design, the lowest cost, and the highest efficiency.

## 3. Preliminaries

### 3.1. Load Forecasting

Load forecasting calculates future load consumption based on various historical data and information available as load patterns. The forecasting for different time horizons is essential for additional operations within the utility. These horizons are classified as very short-term load forecasting (VSTLF), STLF, medium-term load forecasting (MTLF), and long-term load forecasting (LTLF). The duration of VSTLF varies from a few minutes to a few hours ahead and mostly 24 h. The major factors affecting this type of LF are weather and special events, such as festivals and special TV programs. It is mainly used for load shading policies, supply and demand gap investigation, power purchasing and selling, switching operation, generation scheduling, and fuel requirements [33].

STLF duration varies from one day to a few weeks ahead. It is essential for planning to start-up and shut down the protocol for generating unit operation, load management, spinning reserve, carrying out load flow, improvement of transmission and distribution system, unit commitment calculation, loss reduction, and a maintenance schedule of the distribution system.

MTLF involves a horizon from a few days to a few months ahead; this is preferred for balance sheet calculation and risk management. It suits outage and maintenance planning and load switching operation, renovation and modernization of existing generation plants, calculating the capital cost of different generation options, calculating various operational costs, and calculating power to sell or buying with the neighboring power system.

LTLF leads time is measured in months, quarters, or even years. Many factors affect it, such as economic factors, population factors, and renewable integration. It concerns determining the power plant’s future site and capacity and developing the newer ones and the national grid expansion. The LTLF is preferred for carrying out demand-side management, fuel mix decisions, investment decisions in the generation and transmission systems, and preparing the maintenance schedule of generating units. It is worth mentioning that the extended period is better to schedule resources in the marketplace. LTLF is based on the annual trends to ensure that load can be met in the years and decades to forecast regarding transmission line planning and generation planning. Trend analysis in LTLF extends past electricity demand rates into the future using techniques that range from hard-drawn straight lines to complex computer software that produces curves trend analysis. It focuses on the changes in electricity demand and uses them to predict future growth in electricity demand. The advantage of trend analysis is that it is simple, quick, and inexpensive to perform. The disadvantage of the trend forecast is that it produces only the future electricity demand without giving feedback. It does not analyze why electricity demand behaves the way it does [34].

### 3.2. STLF

It is essential to develop the STLF model, requiring a more straightforward structure and faster speed convergence. Besides, it is necessary to make some explicit assumptions to simplify the process of STLF. It starts with collecting and managing the high-quality data on the site of measurement, then using the best archives of weather data from the government agencies. The best high-resolution topography and land cover information can be added, especially in wind and PV solar energy forecasting. Then the best model is picked that fits the data set. After that, use the model and analyze the data and forecast the system. Finally, verification and benchmarking are handled by comparing the forecasted variable to reality.

Many factors control accurate load forecasting in power systems: weather influence, time factors, customer classes, economic and environmental class, unforeseeable random events, and historical data. The electrical load has an obvious correlation to weather. Some essential variables are responsible for load fluctuations such as humidity, wind speed, and direction, thunderstorm, fog, snow, rain, global radiation, sunshine, and dew point; of course, not all the weather parameters are the same importance. If the winter is colder than the normal one and the summer, then the electricity demand is also higher—these weather data needed to be available in real-time observation. Besides, refinement to weather inputs could lead to substations cost-saving and more efficient use of resources. For example, climate change affects infrastructure and load integration of renewables such as solar and wind resources availability. The average weather variability is very high and the noise often swamps the climate signal in the LTLF. Climate change is considered a scientific reality and a political minefield. However, variability and uncertainty are intrinsic to weather and the energy consumption relationship and their effects on the power and power markets.

Time factors are represented by the day of the week, the hour of the day, and holidays, seasonal variation, daily variation, holidays, and vacation. Besides, there is less demand at midnight because of fewer human activities. Customer load demands can be broadly divided into three groups (industrial, residential and commercial). Whereas the economic factors such as electricity price, population change, and demand-side management. The environmental factors such as service are demographic (rural and urban). Some special events include starting the school year, football matches, and quarantine because of the coronavirus and the beginning of the school year. There are four broad categories for a load: domestic loads such as lighting and fans. It has the most constant annual growth rate and the most seasonal fluctuation. The seasonal variation is because of the widespread use of weather-sensitive devices such as air conditioners and space heating. The industrial loads are considered as base loads; it is little weather dependent variables. The commercial loads, such as shops and electrical appliances used in commercial establishments are characterized by seasonal variation. Agricultural loads: This type of load is required to supply water for irrigation using suitable pumps driven by electric motors. The system’s load is constantly changing; variation is more at the consumer ends than at the substation transformer. There is no such term as “steady-state load”.

The electrical data is collected and analyzed based on the actual operating supervisory control and data acquisition (SCADA) and meter readings. Thus it is vital to get clean operational data to understand the actual performance. Indeed, the use of more data improves load forecasting. Hence, utilities should work on expediting and transform the data to represent the system best. It leads to a smooth operation of the grid. The available tribal knowledge is based on past real-life experience. However, the data is always going to be old. Although historical data plays a significant role in load forecasting, management becomes a slave to the historical data and trends rather than the new business reality in the worst scenario. There is no pledge that the conditions in the past will reoccur shortly. It is not reasonable to factor in unique or unexpected events and externalities. Turning a large volume of complete data into actionable information. The data set can be used to make better decisions based on descriptive (what happened?), diagnosis (Why did it happen?), predictive (What will happen? And What should be done?).

STLF is usually done 24 h ahead of when the weather forecast for the following day becomes available. The STLF is very critical to the supplier’s utility. It shows how many generators are in operation, which means shut up some units when the forecasted load is low and start-up some units when the forecasted load is high. This load comprises four parts: baseload $\left(L_{B}\right)$ is the portion of the load that is found to be dependent on overall economic activity and climate conditions for a specified area; weather dependent load $\left(L_{W}\right)$; particular event load $\left(L_{C}\right)$ is the load demand added to special events or a religious and social occasion and the complete random term $\left(L_{R}\right)$ such as noise. Hence, the total demand D can be represented as

(1) $$D=L_{B}+L_{W}+L_{C}+L_{R}$$

## 4. Data Collection

The electrical data is collected and analyzed based on the actual operating supervisory control and data acquisition (SCADA) and meter readings. Thus it is vital to get clean operational data to understand the actual performance. Indeed, the use of more data improves load forecasting. Hence, utilities should expedite and transform the data to represent the system best; this leads to a smooth operation. In modeling load forecast, there are different day type patterns. The user defines the day type for days with the same energy consumption paradigm, such as the national holidays, religious holidays, and social holidays. The load series is complex and exhibits several levels of seasonality. The load depends on the load at the previous hour and simultaneously on the same denomination day. The accumulated connected load is the sum of the continuous rating of the entire load-consuming apparatus connected to the system. Simultaneously, the system’s maximum demand is the greatest of all demands that have occurred during a specified period (daily, monthly, and yearly). A simple way to save on the peak day includes postponing home appliances until the peak day or peak hour is over. After wrangling these data, it is used to forecast daily power availability. Actual load data for more than a year is obtained from one of the leading electricity companies in Jordan. Weather data is collected from the weather station across Jordan.

A new forecasting model is developed that is dynamic and can seem future forecasting downtime. It starts with collecting some data, fitting the model, and then using it to know the forthcoming response’s value. The primary input data set is based on the historical consumption, calendar, day type, weather forecast, and observations. A dimensional reduction is necessary to be considered when the data set is selected. One way is the empirical deduction; it is based on the highly correlated variables and thus redundant, besides these data not correlated with energy consumption. Another way is the principal component analysis. It is based on seeking the maximization of the variance between the dimensions of the data set. Data should not exhibit multicollinearity.

STLF is conducted using the load data from 1 January 2019 to 31 December 2019. as a training set. As training set and the target set is 1 January 2020 to 7 January 2020. The load data is recorded every hour. Electrical consumption varies depending on many parameters, seasonality climate, electrical prices, and exceptional events. The scenarios that are typically obtaining a good balance between accuracy and simplicity for selecting the optimal set of input neurons are:

1. Temperature (${}^{\circ }$C), $T_{1}\left(k\right)$;
2. Hour of the day (00:00/12:00 a.m.–23:00/11:00 p.m.), $T_{2}\left(k\right)$;
3. Day type such as workday, weekend and holiday (−1 for work day, −2 for holiday, 0 for Friday and Saturday), $T_{3}\left(k\right)$;
4. Day of the week (1/Sunday–7/Saturday), $T_{4}\left(k\right)$;
5. Week number in a year (1, 2, …, 52), $T_{5}\left(k\right)$.
6. Month number in a year (1/January, 2/February, …, 12/December), $T_{6}\left(k\right)$.

This chosen structure to build the load forecasting model is the one that gives the best performance in terms of accuracy, reliability, efficiency, simplicity, complete control over power prediction, minimizes the foretasted error, and ensures success based on the trusted partnership industry. Two different cases are studied: hourly daily load and total daily load. The total daily load can be defined as the sum of the baseload and weather-dependent components. It is given as in Equation (2):

(2) $$W_{total}\left(d\right)=\sum_{h=1}^{24}W(h,d)$$

## 5. Methodology

### 5.1. Multi-Layer Perceptron Neural Networks (MLP)

**Figure 1:**
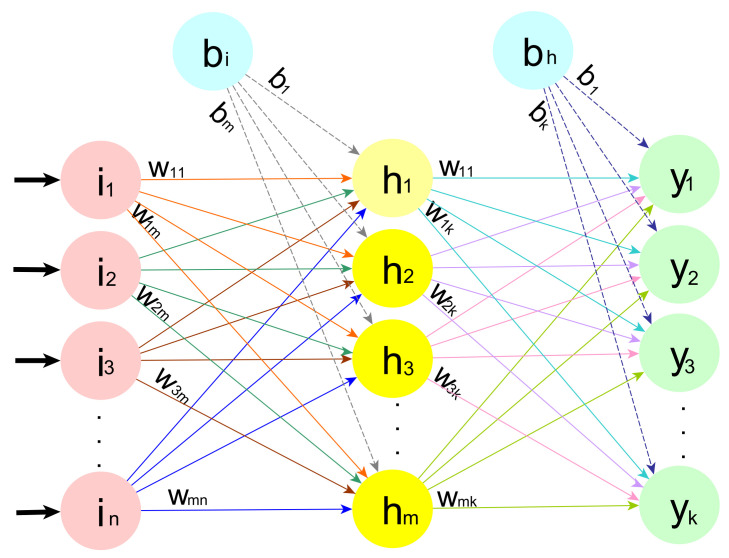
ANN architecture.

The artificial neural network is a sub-field of machine learning where the human brain neural network structure inspires the algorithm. the data are collected and trained to recognize the patterns in this data and then predict the output for a new set of similar data [35,36,37]. The MLP is a well-known type of FNNs. It comprises layers of neurons; these neurons are the core processing units of the network. There are three layers, the input layers that receive the input and the output layers that predict the final output, whereas the hidden layers are located between the two layers. These hidden layers perform most of the computation required by the network. The number of hidden layers depends on the complexity of the problem. Researchers commonly, use a single layer by default. Figure 1 shows a default MLP with input layer compromises *n* neurons, a single hidden layer compromises *m* neurons and an output layer compromises *k* neurons.

The MLP represents a directed connected graph in which each hidden neuron is connected with *n* connection weight. In addition, a bias weight enters each hidden neuron. Two primary processes are performed inside each hidden neuron: summation and activation. The output of the summation process of neuron *j* is performed using Equation (3). After the summation is accomplished, the output is mapped using a particular function called transfer or activation function. The activation function is performed using Equation (4).

(3) $$Sum_{j}=\sum_{i=1}^{n}w_{ij}*in_{i}+b_{j}$$

where $w_{ij}$ represents the connection weight between the input *i* in the input layer and the neuron *j* in the hidden layer, $b_{j}$ is the bias *j* to the hidden neuron *j*.

(4) $$y_{j}=f\left(sum_{j}\right)$$

where $y_{j}$ is the output neuron *j*; j=1,2,…m; *f* is the sigmoid function and computed as in Equation (5)

(5) $$f\left(sum_{j}\right)=\frac{1}{1+e^{-sum_{j}}}$$

After collecting the outputs of all the hidden neurons, the final outputs $Y_{j}$ are computed based on the summation and activation functions as shown in Equations (6) and (7).

(6) $$Sum_{j}=\sum_{i=1}^{m}w_{ij}*y_{j}+b_{j}$$

where $w_{ij}$ represents the connection weight between the hidden neuron *i* in the hidden layer and the neuron *j* in the output layer, $b_{j}$ is the bias *j* to the output neuron *j*.

(7) $$y_{j}=f\left(sum_{j}\right)$$

where $Y_{j}$ is the final output *j*; j=1,2,…k; *f* is the same sigmoid function used in Equation (5).

### 5.2. GWO

A metaheuristic is a developed optimization algorithm with a high-level problem independent framework [38,39,40]. The best solution in the metaheuristic algorithm is found out of all possible solutions of an optimization. GWO is one of the metaheuristic algorithms proposed by Mirjaliali Mohammad and Lewia in 2014. GWO is a well-established swarm intelligence methodology that has been utilized in a wide range of applications with promising achieved performance. The idea of GWO is inspired by the grey wolves’ hierarchy in nature and their strategy in hunting prey. Gray wolf (Canis lupus) is a sizeable canine animal; Its running speed is around 50–60 km/h. The lifespan in the wild is about 6 to 8 years. It belongs to the Canidae biological family of dogs like carnivorans. The weight is around 40 kg (M) and 37 kg (F). The length is approximately (105 cm–160 cm) (M) and (80 cm–85 cm) (F). The height is (40 in–63 in.) (M) and (31 in–33 in) (F). It lived in highly organized packs and is considered one of the most successful animals on earth. It is well known as loyal to the pack members and ability to work together in a pack. The average pack size ranges from 5–12, with different ranks of wolves in a pack (Alpha, Beta, Delta, and Omega). The leader of the pack is Alpha. It is responsible for making decisions about hunting, sleeping places, and time to wake up. The second level is Beta. It is considered the best candidate to be Alpha. Beta helps Alpha in decisions making and other activities such as giving feedback to Alpha. Delta is responsible for any danger facing the pack and providing food. Whereas Omega is the lowest rank in the pack, it plays the scapegoat or victim rule. Other ranks blame it for their mistakes or faults. Over that, it is the last one allowed to eat. GWO algorithm mimics the hunting mechanism and leadership of the gray wolves. The optimization mechanism is based on searching for the prey, tracking, chasing, and approaching the prey. Then, pursuing, encircling, and harassing the prey. Finally, attaching the prey. Whitetail, mule deer, moose, and caribou are considered the main prey and the desired target for the gray wolf.

**Figure 2:**
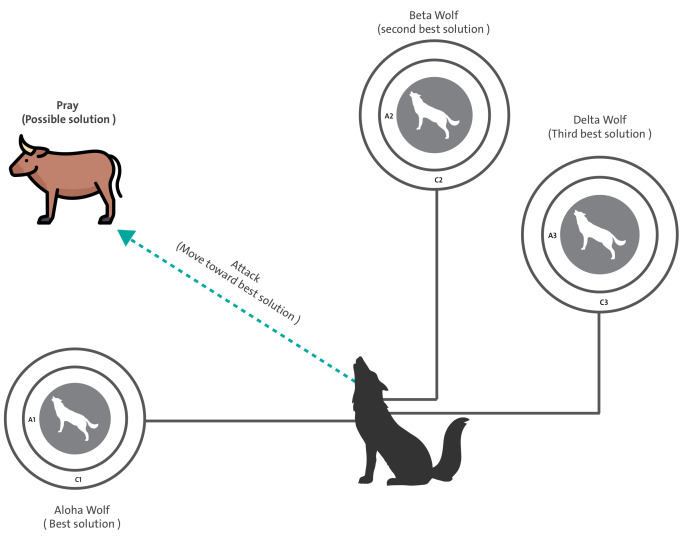
Illustration of the position update strategy for ω wolf based on the position of α and β leaders.

Briefly, GWO arranges the wolves in the pack based on their fitness into four levels: α is the best candidate solution found so far, β the second-best candidate solution found so far, δ is the third-best candidate solution found so far, and ω are the remaining solutions in the pack (see Figure 2). The hunting behavior of wolves is modeled by emulating two processes: encircling prey and then hunting it.

#### 5.2.1. Encircling Prey

The distance between each attacking wolf and the prey can be formulated as in Equation (8).

(8) $$\vec{D}=\left|\vec{C}.{\vec{X}}_{P}\left(t\right)-\vec{X}\left(t\right)\right|$$

(9) $$\vec{C}=2{\vec{r}}_{2}$$

where ${\vec{X}}_{P}\left(t\right)$ represents the vector of the prey’s position in the search space. $\vec{X}$(*t*) represents the vector of a wolf’s position in the search space, and *t* is the current iteration of the optimization process. *C* is calculated as in Equation (9), and ${\vec{r}}_{2}$ is a vector in the interval [0, 1].

#### 5.2.2. Hunting Prey

An attacking wolf changes its position and becomes closer to prey to hunt it. Based on the distance between a wolf and prey as computed in Equation (8), the next position of a wolf is formulated as in Equation (10).

(10) $$\vec{X}\left(t+1\right)={\vec{X}}_{P}\left(t\right)-\vec{A}.\vec{D}$$

(11) $$\vec{A}=2\vec{a}.{\vec{r}}_{1}-\vec{a}$$

where $\vec{a}$ linearly decreases from 2 to 0 over the iterations, and $r_{2}$ is a random vector in the range [0, 1].

According to the natural wolves hierarchy in the pack, the wolves in the ω group have less knowledge in determining the position of the prey compared with the best three wolves α, β, and δ. Therefore, the distances between each wolf in the pack and the first three best wolves are computed as in Equation (12).

(12) $$\begin{array}{cc} {\vec{D}}_{\alpha } & =|{\vec{C}}_{1}.{\vec{X}}_{\alpha }-\vec{X}|\\ {\vec{D}}_{\beta } & =|{\vec{C}}_{2}.{\vec{X}}_{\beta }-\vec{X}|\\ {\vec{D}}_{\delta } & =|{\vec{C}}_{3}.{\vec{X}}_{\delta }-\vec{X}| \end{array}$$

The computed distances from Equation (12) are used to change the position of a wolf in the search space using Equations (13) and (14).

(13) $$\begin{array}{cc} {\vec{X}}_{1} & ={\vec{X}}_{\alpha }-{\vec{A}}_{1}.{\vec{D}}_{\alpha }\\ {\vec{X}}_{2} & ={\vec{X}}_{\beta }-{\vec{A}}_{2}.{\vec{D}}_{\beta }\\ {\vec{X}}_{3} & ={\vec{X}}_{\delta }-{\vec{A}}_{3}.{\vec{D}}_{\delta } \end{array}$$

(14) $$\vec{X}\left(t+1\right)=\frac{{\vec{X}}_{1}+{\vec{X}}_{2}+{\vec{X}}_{3}}{3}$$

By repeating the encircling and hunting processes, the best position of prey can be determined. Algorithm 1 shows the pseudocode of the GWO algorithm.
**Algorithm 1** GWO pseudo-code.Set the maximum number of iterations *T* Initialize the swarm $X_{i}$ (i = 1, 2, …, n) Initialize *a*, *A*, and *C* Compute the fitness values of wolves $X_{\alpha }$ = the first-best solution $X_{\beta }$= the second-best solution $X_{\delta }$ = the third-best solution   **while** (*t* < *T*) **do**   **for** each wolf in the swarm **do**    Update the position of the current wolf by Equation (12)   **end for**   Update *a*, *A*, and *C*   Compute the fitness values of all wolves   Update $X_{\alpha }$, $X_{\beta }$ and $X_{\delta }$   t = t+1 **  end while**  Return $X_{\alpha }$


### 5.3. GWO for Learning Neural Networks (fNN-GWO)

In the literature, many swarm intelligence algorithms have been utilized for training the neural network. GWO is a well-regarded algorithm that proved its success in solving many optimization problems in different applications. It could outperform other algorithms such as the particle swarm algorithm (PSO) [41]. The widespread usage of GWO returns to its impressive properties. GWO has a specialized mathematical method that is inspired by the hierarchical social system of grey wolves. This allows the solutions to change their positions in the n-dimensional space simulate the chasing and encircling techniques of grey wolves around prey. GWO has few parameters and can balance exploration and exploitation during the optimization process. This causes to control the convergence behavior of the optimizer and alleviates the premature convergence. In addition, its ease of use, flexibility, and scalability encouraged us to use it for optimizing the training process of the neural network.

The primary task of GWO is to find the optimal set of network connections (weights and biases). For the hidden nodes in the MLP, there is no standard way to determine their number. Therefore, GWO uses a fixed structure for MLP with the number of nodes determined based on Equation (15):

(15) m=2×d+1

where *m* is the number of neurons, *d* is the number of input data (features). Therefore, the total number of weights and biases *n* is found based on Equation (16).

(16) n=d×m+2×m+1

**Figure 3:**
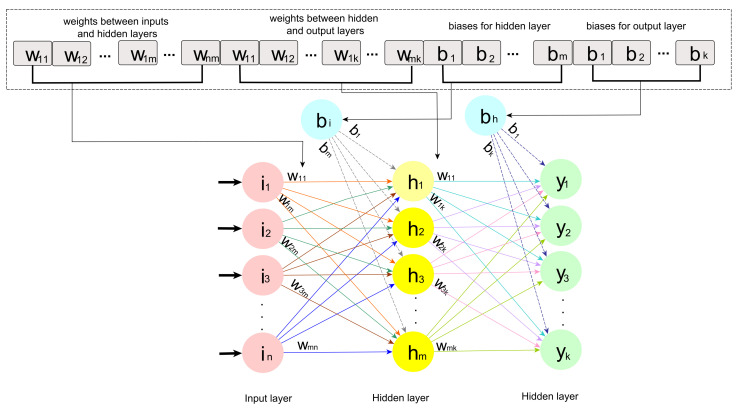
Mapping a GWO individual into MLP network architecture.

To use GWO for optimizing weights and biases, the individuals (grey wolves) are used to represent them. Each individual is represented by a 1d-array or a vector with real numbers for their components. The individual representation and its mapping to an MLP network are illustrated in Figure 3. MLP training can be performed by GWO by integrating the GWO operators with the MLP network.

**Figure 4:**
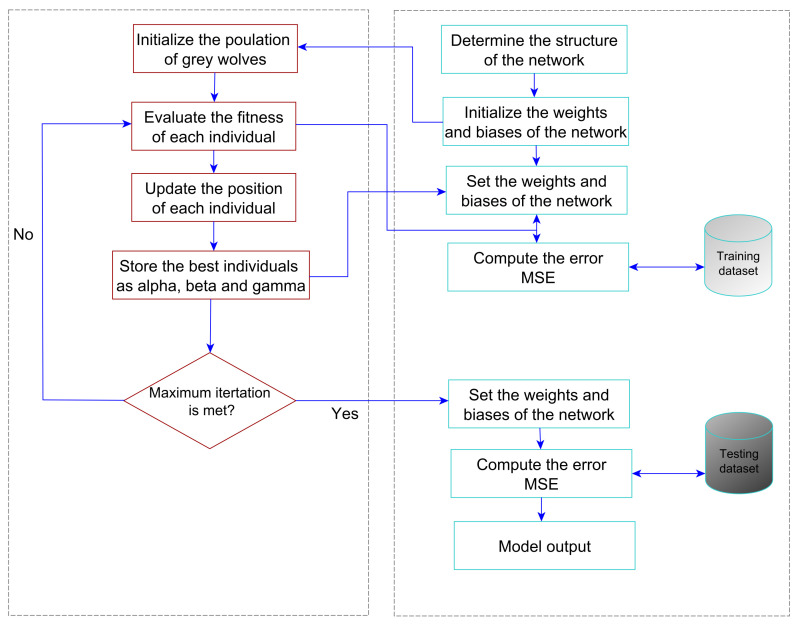
Flowchart of the proposed learning model (GWO-MLP).

The flowchart of the proposed learning method is shown in Figure 4. The training process can be summarized in the following steps:

- Initialization: This requires specifying the MLP structure by determining the number of neurons *m* and the total number of weights and biases *n*. Then *N* individuals are initialized to generate random numbers of weights and biases.
- Fitness evaluation: Based on a specified fitness function, the individuals are evaluated to give each one a fitness value. In this paper, the mean squared errors MSE are used as shown in Equation (17).
  (17) $$MSE=\frac{1}{k}\sum_{i=1}^{k}{\left(y_{i}-\hat{y_{i}}\right)}^{2}$$
  where $y_{i}$ is the actual output of $i_{th}$ training instance; $\hat{y_{i}}$ is the predicted output of $i_{th}$ training instance, *k* is the total number of the training instances.
- Update: In this step, the update procedure for the repositions of grey wolves in the search space is applied
- Termination: These steps are repeated until a specified stopping condition is satisfied (the maximum number of iterations is reached).

After training the MLP using the training instances, the best set of weight connections and bias set is specified. This global best set is applied then on the testing part of the dataset. Therefore the MLP can serve as a predictive tool [42].

The aforementioned steps make the GWO generate a set of new MLP networks until it determines the best network at the end. The steps of computing MSEs and improving the MLPs continue until the maximum iterations are reached. The average MSE is computed when classifying all training instances in the dataset for each MLP network in the proposed GWO-based trainer. Therefore, the computational complexity is O(ntd) where *n* is the number of random MLP networks, *t* is the maximum iterations, and *d* is the number of training instances in the dataset.

## 6. Experiments and Results

In this section, the proposed GWO-based (fNN-GWO) load forecasting approach for training MLP networks is evaluated using the collected dataset that is mentioned in the data collection section. For all experiments, the MATLAB R2015b is used to implement the proposed methodology and other algorithms.

As was mentioned in the data collection section, the data that was collected from 1 January 2019 to 31 December 2019 (for one year) is used for training the models, and the data was collected from 1 January 2020 to 30 April 2020 (4 months) is used for testing the model. Furthermore, The number of training data records is 8760 instances for hourly daily load consumption, and 2880 instances in testing data. For total daily load consumption, the number of instances in the training dataset is equal to 360 records, and the number of instances in the testing dataset is equal to 120 records. The fNN-GWO is compared with five well-known nature-inspired algorithms: genetic algorithm (fNN-GA), particle swarm optimization (fNN-PSO), ant colony optimization (fNN-ACO), evolution Strategy (fNN-ES), and whale optimization algorithm (fNN-WOA), which have been used in the previous works to train the neural network. In addition, the proposed method is compared with linear regression (LR), which is considered the most common method was used for modeling regression problems [43,44].

### 6.1. Evaluation and Experimental Settings

To evaluate the performance of the proposed model, different evaluation measures have been adopted, including mean squared errors (MSE), errors difference (ED), mMean absolute error (MAE), and the root mean squared error (RMSE).

- MSE: It shows the deviation of the predicted errors that show how much the predicted points are close to the target line, represented by Equation (18).
  (18) $$MSE=\frac{1}{n}\sum_{i=1}^{n}{\left(y_{i}-\hat{y_{i}}\right)}^{2}$$
- ED: It shows the deviation of the predicted errors that show how much the predicted points are close to the target line, represented by Equation (18).
  (19) $$ED=\sqrt{\sum_{i=1}^{n}{\left(y_{i}-{\hat{y}}_{i}\right)}^{2}}$$
- MAE: It is the average of the magnitude of the predicted errors, presented by Equation (20).
  (20) $$MAE=\frac{1}{n}\sum_{i=1}^{n}\left|y_{i}-{\hat{y}}_{i}\right|$$
- RMSE: It shows the deviation of the predicted errors that show how much the predicted points are close to the target line, represented by Equation (21).
  (21) $$RMSE=\sqrt{\frac{\sum_{i=1}^{n}{\left(y_{i}-{\hat{y}}_{i}\right)}^{2}}{n}}$$

### 6.2. The Effect of the Number of Layers in the Feed-Forward Neural Network

Table 1 illustrates the results of the error forecasting for hourly daily load consumption. Five different experiments were performed, where each experiment corresponds to the number of layers. As shown in the Table, the feed-forward neural network got the best results in all measures when the number of layers was four. For instance, in terms of MSE, the result was 77,611.56, which is better than the nearest layer by 5034.07. As for the other measures, the results were 14,914.43, 221.28, and 277.91 for ED, MAE, and RMSE, respectively. While the second-best was achieved when the layer equal two with 82,645.63, 15,398.16, 228.91, and 286.93 for MSE, ED, MAE, and RMSE, respectively.

Further, the total daily load consumption results are depicted in Table 2. The feed-forward neural network, when the number of layers is equivalent to five, outperforms the other layers in terms of all measures. For example, the MSE is equal to 18,601,703.82, which is better than the best-second with 1,975,658.08. As for ED, MAE, and RMSE, the results were 47,045.26, 3672.95, and 4294.62, respectively. The order of the finest layers after layer-five in terms of all measures was three, two, four, and one, respectively.

### 6.3. Comparative Analysis

#### 6.3.1. A Comparison between fNN-GWO and LR

**Figure 5:**
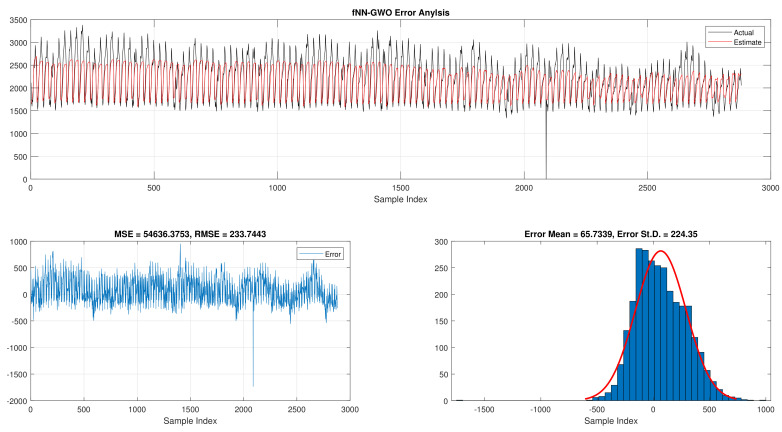
Error analysis of the hourly daily load consumption with fNN-GWO best results.

**Figure 6:**
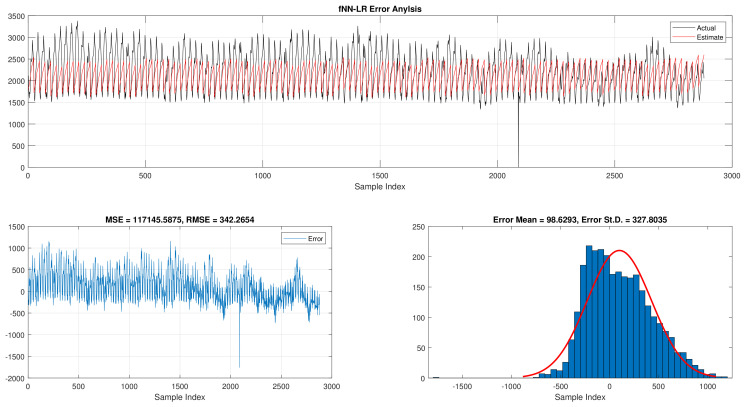
Error analysis of the hourly daily load consumption with LR best results.

When comparing the best results for fNN-GWO in the hourly daily load consumption with LR as seen in Table 3. The fNN-GWO exceeds the LR in all measures with a difference of 39,534.03, 3453.46, 49.79, and 64.36 for MSE, ED, MAE, and RMSE, respectively. The error analysis of the hourly daily load consumption for both methods can be found in Figure 5 and Figure 6. The Figures show the best run for fNN-GWO and LR and the actual and estimated results and error. Besides, the best run for MSE and RMSE results were shown.

**Figure 7:**
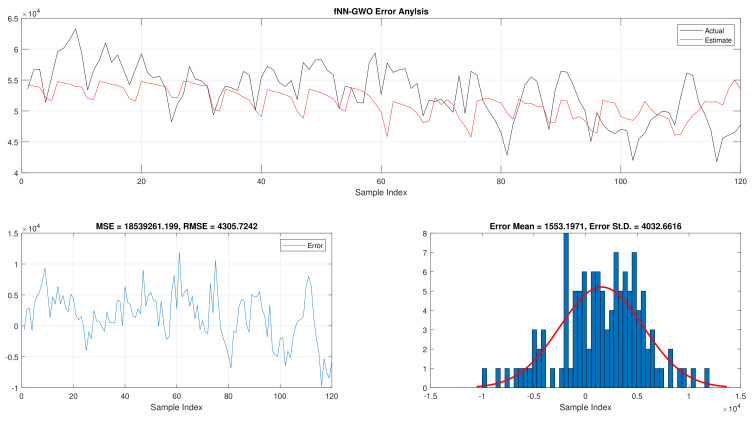
Error analysis of the total daily load consumption with fNN-GWO best results.

**Figure 8:**
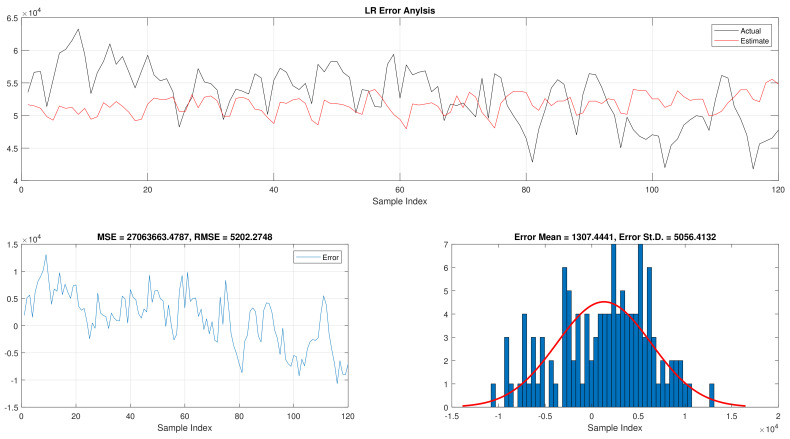
Error analysis of the total daily load consumption with LR best results.

The total daily load consumption results for fNN-GWO and LR can be found in Table 4. Similar to the previous experiments, the fNN-GWO outperforms the LR in all measures. The superiority of fNN-GWO compared to LR was better by 8,461,959.66, 9942.81, 715.1, and 907.65 for MSE, ED, MAE, and RMSE, respectively. The error analysis of the total daily load consumption for both methods can be found in Figure 7 and Figure 8.

#### 6.3.2. A Comparison between fNN-GWO and Other Meta-Heuristics

**Figure 9:**
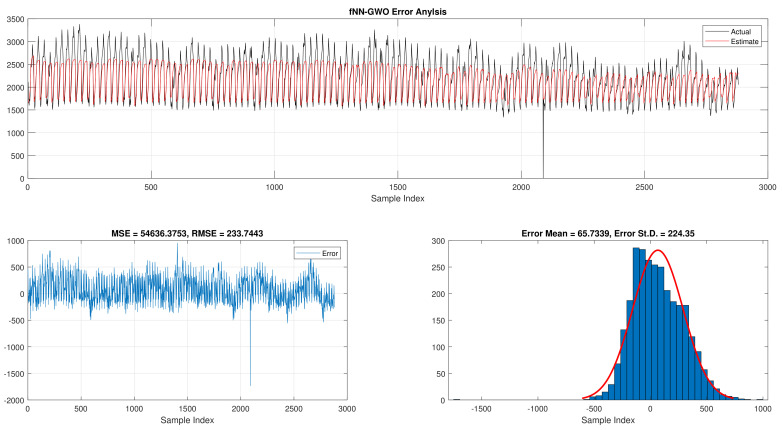
Error analysis of the hourly daily load consumption with fNN-GWO best results.

**Figure 10:**
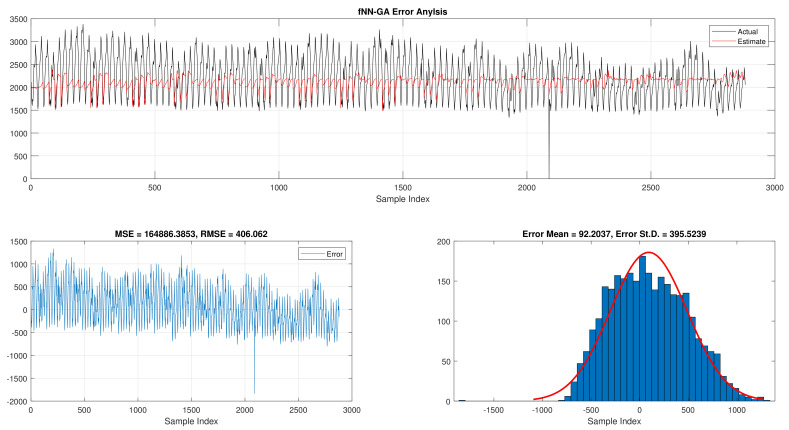
Error analysis of the hourly daily load consumption with fNN-GA best results.

**Figure 11:**
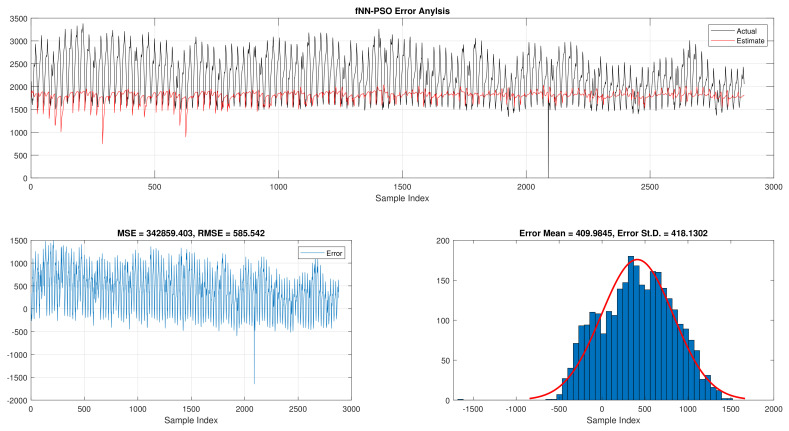
Error analysis of the hourly daily load consumption with fNN-PSO best results.

**Figure 12:**
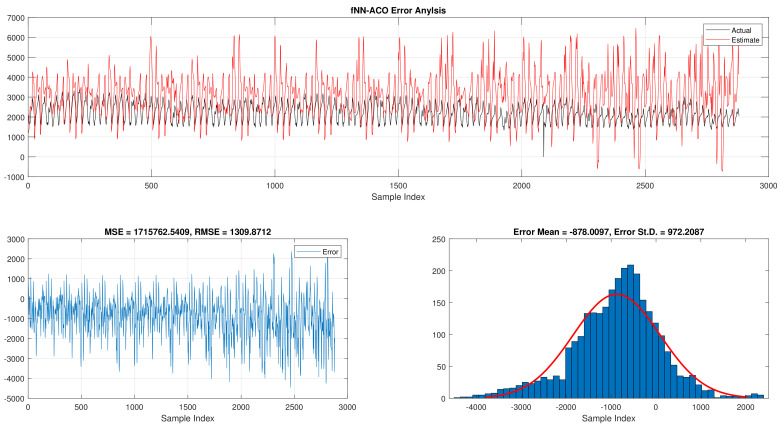
Error analysis of the hourly daily load consumption with fNN-ACO best results.

**Figure 13:**
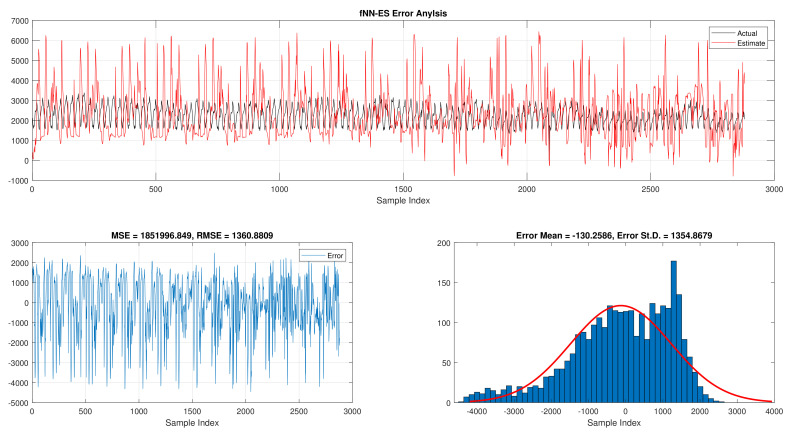
Error analysis of the hourly daily load consumption with fNN-ES best results.

**Figure 14:**
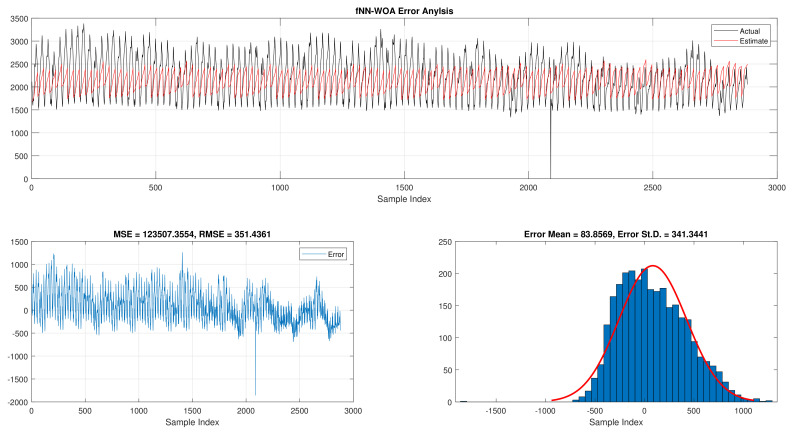
Error analysis of the hourly daily load consumption with fNN-WOA best results.

The comparison of fNN-GWO and other meta-heuristics for hourly daily and total daily load consumption is presented in this subsection. Table 5 illustrates the results of fNN-GWO and other meta-heuristics for the hourly daily load consumption. As shown, the fNN-GWO gained the best results in all measures, followed by fNN-WOA, fNN-GA, fNN-PSO, fNN-ACO, fNN-ES, and fNN-WOA, respectively. The difference between fNN-GWO and the nearest meta-heuristic (fNN-WOA) were 173,811.71, 11,069.45, 167.11, 277.91, and 206.27 for MSE, ED, MAE, and RMSE, respectively. The error analysis of the hourly daily load consumption for fNN-GWO, fNN-GA, fNN-PSO, fNN-ACO, fNN-ES, and fNN-WOA methods can be found in Figure 9, Figure 10, Figure 11, Figure 12, Figure 13 and Figure 14, respectively.

**Figure 15:**
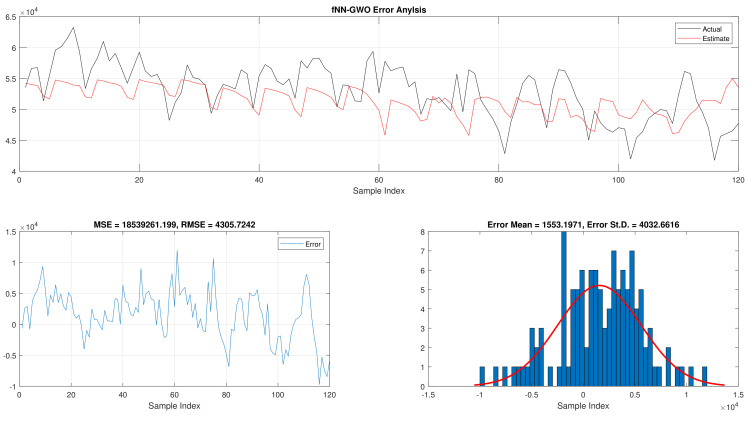
Error analysis of the total daily load consumption with fNN-GWO best results.

**Figure 16:**
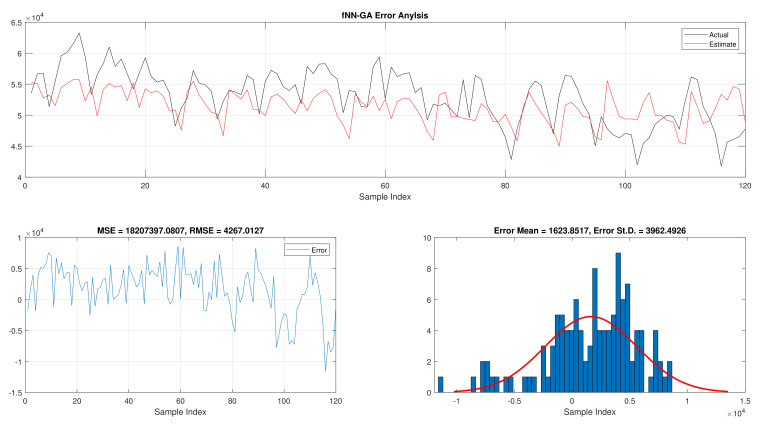
Error analysis of the total daily load consumption with fNN-GA best results.

**Figure 17:**
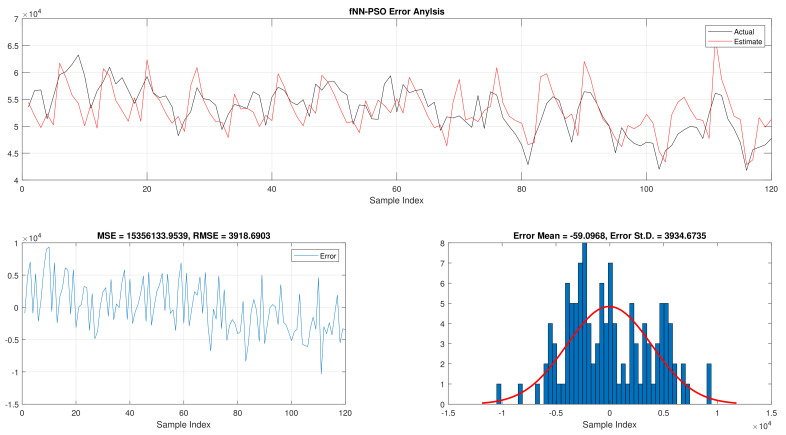
Error analysis of the total daily load consumption with fNN-PSO best results.

**Figure 18:**
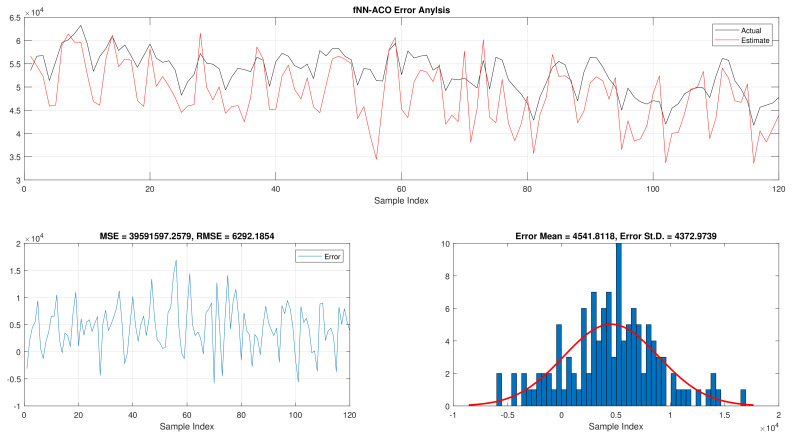
Error analysis of the total daily load consumption with fNN-ACO best results.

**Figure 19:**
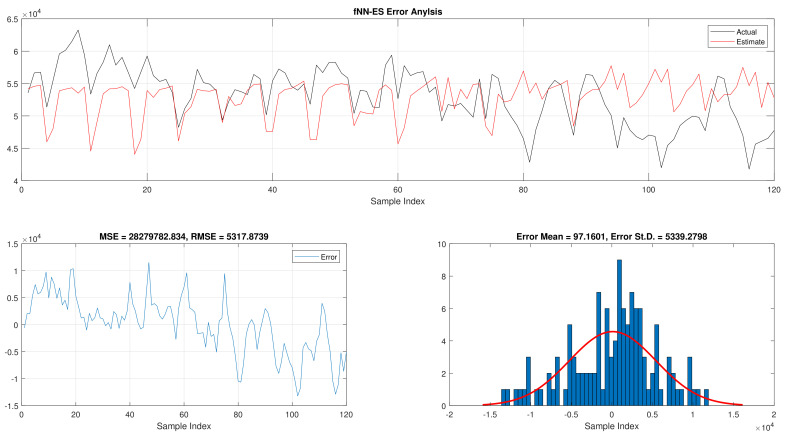
Error analysis of the total daily load consumption with fNN-ES best results.

**Figure 20:**
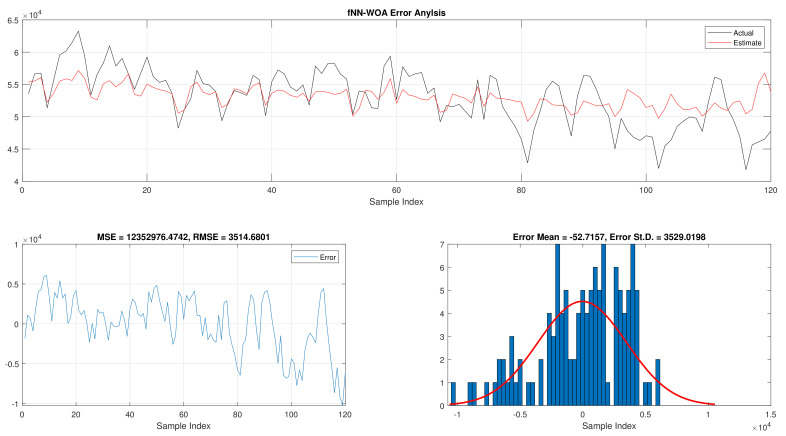
Error analysis of the total daily load consumption with fNN-WOA best results.

The results of the total daily load consumption are portrayed in Table 6. Like previous experiments, the fNN-GWO exceeds the other meta-heuristic algorithms with all measures, followed by, fNN-GA, fNN-PSO, fNN-ACO, fNN-ES, and fNN-WOA. The difference between fNN-GWO and the closet one (fNN-GA) for MSE, ED, MAE, and RMSE were 5,924,502.24, 6709.77, 391.91, and 612.52, respectively. Figure 15, Figure 16, Figure 17, Figure 18, Figure 19 and Figure 20 describes the best run for fNN-GWO, fNN-GA, fNN-PSO, fNN-ACO, fNN-ES, and fNN-WOA, respectively.

The overall experiments show the superiority of fNN-GWO compared with LR and other meta-heuristic algorithms for prediction and forecasting the load consumption of both hourly and total daily.

## 7. Conclusions

Forecasting is a risky art. However, it is needed for better future planning. Electric load forecasting is the science of the utility company’s pre-most economical amount of electric power. It should be timely, as accurate as possible, reliable, and in some meaningful units. This study helps minimize the gap between the demand and the supply of power to avoid a shortage of surplus in the power supply. Enhanced planning, optimized operation, improved forecasting, increased efficiency, and reduced cost are considered the main goals of the electrical power system operator. This research proposed an optimized multi-layered feed-forward neural network using the recent Grey Wolf Optimizer (GWO). The problem of power forecasting is formulated as an optimization problem. The experimental results are compared with popular optimization methods and show that the proposed method provides very competitive forecasting results.

In the future work of forecasting studies, it needs to include regulator changes and reliability standards. One of the significant challenges in load forecasting studies is the range of planned actions or things expected to occur besides the lack of data science.

## Figures and Tables

**Table 1:** Error forecasting results for hourly daily load consumption, using a different number of layers feed forward neural network.

No of Layeres	MSE	STD	ED	STD	MAE	STD	RMSE	STD
1	105,221.21	13,320.04	17,376.83	1096.92	256.26	16.15	323.80	20.44
2	82,645.63	10,894.69	15,398.16	1008.89	228.91	13.54	286.93	18.80
3	86,044.61	12,596.42	15,703.18	1163.57	236.17	17.38	292.61	21.68
4	**77,611.56**	11,053.10	**14,914.43**	1096.01	**221.28**	17.01	**277.91**	20.42
5	92,252.12	187,77.97	16,224.35	1652.13	238.92	25.82	302.32	30.79

**Table 2:** Error forecasting results for total daily load consumption, using a different number of layers feed forward neural network.

No of Layeres	MSE	STD	ED	STD	MAE	STD	RMSE	STD
1	22,042,477.63	3,301,886.28	51,306.17	3767.44	3939.73	352.68	4683.59	343.92
2	20,673,304.95	3,621,876.15	49,631.00	4417.21	3883.22	404.57	4530.67	403.23
3	20,577,361.90	2,392,275.99	49,616.17	2890.49	3864.96	215.40	4529.32	263.86
4	21,331,215.00	2,209,367.70	50,532.48	2627.64	3946.97	264.68	4612.96	239.87
5	**18,601,703.82**	3,639,488.12	**47,045.26**	4588.44	**3672.95**	382.96	**4294.62**	418.87

**Table 3:** A comparison between fNN-GWO error forecasting results for hourly daily load consumption, and LR.

Algorithms	MSE	ED	MAE	RMSE
fNN-GWO	**77,611.56**	**14,914.43**	**221.28**	**277.91**
LR	117,145.59	18,367.89	271.07	342.27

**Table 4:** A comparison between fNN-GWO error forecasting results for total daily load consumption and LR.

Algorithms	MSE	ED	MAE	RMSE
fNN-GWO	**18,601,703.82**	**47,045.26**	**3672.95**	**4294.62**
LR	27,063,663.48	56,988.07	4388.05	5202.27

**Table 5:** A comparison between fNN-GWO error forecasting results for hourly daily load consumption, and other meta-heuristics methods.

Algorithms	MSE	STD	ED	STD	MAE	STD	RMSE	STD
fNN-GWO	**77,611.56**	**11,053.10**	**14,914.43**	**1096.01**	**221.28**	**17.01**	**277.91**	**20.42**
fNN-GA	52,6763.24	373,468.90	36,670.28	13,839.10	538.75	195.35	683.31	257.88
fNN-PSO	916,129.55	311,586.14	50,560.75	9548.93	762.81	146.94	942.14	177.93
fNN-ACO	5,453,738.12	3,280,577.19	119,974.85	38,192.50	1873.29	645.70	2235.60	711.68
fNN-ES	3,990,513.26	2,141,128.91	104,027.87	27,302.45	1574.91	451.81	1938.44	508.75
fNN-WOA	251,423.27	153,157.68	25,983.85	7374.03	388.31	107.99	484.18	137.41

**Table 6:** A comparison between fNN-GWO error forecasting results for total daily load consumption, and other meta-heuristics methods.

Algorithms	MSE	STD	ED	STD	MAE	STD	RMSE	STD
**fNN-GWO**	**1,860,1703.82**	**3,639,488.12**	**47,045.26**	**4588.44**	**3672.95**	**382.96**	**4294.62**	**418.87**
fNN-GA	24,526,206.02	7,740,171.85	53,754.97	7713.47	4064.86	574.74	4907.14	704.14
fNN-PSO	43,950,581.91	13,230,479.06	71,831.02	11,273.08	5284.14	1019.42	6557.25	1029.09
fNN-ACO	170,763,987.82	125,293,682.53	135,044.06	50,053.09	10,263.39	4254.05	12,327.78	4569.20
fNN-ES	129,578,577.48	64,691,684.36	120,956.12	31,955.64	8753.54	2731.52	11,041.73	2917.14
fNN-WOA	30,921,885.96	18,015,693.11	59,131.57	15,423.04	4446.06	1085.71	5397.95	1407.92

## Abbreviations

The following abbreviations are used in this manuscript:

GWO	Grey Wolf Optimizer
STLF	short-term load forecasting
PSO	Particle swarm optimization
GA	Genetic algorithm
EHO	Elephant Herding Optimization
NN	Neural Network
FFNN	Feed Forward Neural Networks
RBFNN	Radial Basis Function Neural Networks
MSE	Mean Square Error
MAE	Mean Absolute Error
kNN	k-Nearest Neighbors
XGB	eXtreme Gradient Boosting
LGBM	Light Gradient Boosting
MLP	Multilayer Perception
NFL	No-Free-Lunch
VSTLF	Very Short-Term Load Forecasting
MTLF	Medium-Term Load Forecasting
LTLF	Long-Term Load Forecasting
